# Crying the blues: The configural processing of infant face emotions and its association with postural biases

**DOI:** 10.3758/s13414-022-02522-2

**Published:** 2022-06-07

**Authors:** Gianluca Malatesta, Valerio Manippa, Luca Tommasi

**Affiliations:** 1 Department of Psychological, Health and Territorial Sciences–University ‘G. d’Annunzio’ of Chieti-Pescara, Via dei Vestini, 31, I-66100 Chieti, Italy; 2 Department of Education, Psychology, and Communication–University ‘Aldo Moro’ of Bari, Piazza Umberto I, 1, I-70121 Bari, Italy

**Keywords:** Face perception, Mental rotation/visual perception, Perceptual categorization and identification

## Abstract

Several studies have exploited the face inversion paradigm to unveil the mechanisms underlying the processing of adult faces, showing that emotion recognition relies more on a global/configural processing for sadness and on a piecemeal/featural processing for happiness. This difference might be due to the higher biological salience of negative rather than positive emotions and consequently should be higher for infant rather than adult faces. In fact, evolution might have promoted specific adaptations aimed to prioritize the infant face by the attention system in order to foster survival during infancy, a rather long period during which the newborn depends entirely on adults. Surprisingly, no study has yet exploited this paradigm to investigate the processing of emotions from infant faces. In this study, the face inversion paradigm was used to explore emotion recognition of infant compared with adult faces in a sample of adult participants. In addition, the existence of potential differences associated with specific postural biases (e.g., the left-cradling bias) during interactions with infants was explored. The presence of rotational effects for the recognition of both happy and sad infant faces suggests that infant face emotions are predominantly processed in a configural fashion, this perceptual effect being more evident in sadness. Results are discussed in the context of the biological and social salience of the emotional infant face.

## Introduction

It is well established that faces represent one of the most complex and important social stimuli that humans attend to in daily life, with emotional expression representing the vehicle through which social signals are received from and transmitted to other individuals. In order to find out the mechanisms underlying the processing of faces, many studies have exploited the face inversion effect—namely, the phenomenon whereby adult humans perform much better (in terms of accuracy and response times) in recognizing upright faces compared with other mono-oriented objects, and much worse in recognizing inverted (i.e., upside-down or rotated 180°) faces compared with inverted objects (Yin, 1969). It is plausible that this disproportionate effect is due to different mechanisms involved in the processing of upright and inverted faces. In this regard, it has been suggested that a “configural” processing of the spatial relations among the features of a face stimulus—analyzed in a global fashion—is more commonly used for analyzing upright faces (probably due to the extraordinary human expertise in handling this category of stimuli) and a “featural” processing is more commonly used for inverted faces—analyzed in a piecemeal fashion—and most of nonface objects (Maurer et al., 2002). According to this suggestion, recognition of inverted faces entails a switch from configural to featural processing—occurring at around 90°–100° as documented in studies factoring in progressive levels of rotation (e.g., Stürzel & Spillmann, 2000)—and, consequently, a disadvantage in terms of performance compared with upright faces (Carey & Diamond, 1977; Prete, Marzoli et al., 2015b).

Face inversion has also been exploited to investigate the mechanisms underpinning the processing of emotions from faces (Prete, Capotosto, et al., 2015a), showing that inversion has little or no influence on the recognition of happiness, whereas it strongly impairs the recognition of sadness (Calvo & Nummenmaa, 2008). Therefore, it seems that emotion recognition is predominantly based on configural information for processing sad faces and on featural information for happy faces, possibly due to the higher biological salience of negative rather than positive emotions in terms of evolutionary survival (Thompson & Voyer, 2014).

In this study, we investigated the mechanisms of emotion recognition from both adult and infant faces using a face inversion paradigm (180° rotation), to which we added two intermediate angles of rotation (i.e., faces rotated clockwise 90° and 270°). Although the infant face is generally considered a special case in the domain of face perception providing a powerful stimulus for adults (Hahn & Perrett, 2014; Thompson-Booth et al., 2014), to date no study has investigated emotion recognition of infant compared with adult faces using face inversion or face rotation. In fact, several studies have thoroughly explored the many facets of the facial features of infants (i.e., the “baby schema”; Lorenz, 1943) that evolved in order to ensure their survival by eliciting caregiving behaviours from adults (women, especially; (Glocker et al., 2009). In this regard, it has been suggested that the infant face is prioritized by the attention system through the baby schema in order to foster survival and reproductive fitness during infancy (Brosch et al., 2007), albeit no study has yet tried to fully explain whether this mechanism is due either to a configural or to a featural processing of the infant face. Therefore, given that the infant face is a salient stimulus for adults, it is not unreasonable to hypothesize that its emotional recognition relies more on a configural than a featural processing. For instance, a recent study using the inversion effect paradigm has shown that higher maternal sensitivity was associated with a larger use of configural processing for their infants’ body cues (Butti et al., 2018). Although recent research seems to suggest that a common expression-specific emotion recognition mechanism might exist for the processing of both infant and adult specific emotions from faces (Parsons et al., 2011), a general advantage for the processing of infant faces expressing negative emotions has been very recently demonstrated, further confirming the biological salience of this specific valence (Hampson et al., 2021).

Although the proficiency in configural processing of upright faces is considered a stable trait of the adult perceptual system in processing salient stimuli, the timing of its emergence in the early stages of development is still a debated issue. For instance, electrophysiological evidence has highlighted different pathways of cortical activity already functional at birth for the processing of schematic face-like vs. inverted face-like patterns in 1- to 4-day-old attentive newborns (Buiatti et al., 2019). However, it is believed that such an expertise, probably starting from the fourth/fifth month of the child’s age, develops gradually over the first year of life (Cashon & Holt, 2015). It is reasonable to speculate that the ability of the child to process upright faces as adults might go hand in hand with their ability to maintain an upright posture. In other words, switching from a horizontal to a vertical posture might gradually enhance the child’s expertise in the configural processing of upright faces.

Perhaps more importantly to our research question, when adults cradle infants in their arms, they are more likely to be exposed to faces rotated anticlockwise or clockwise, depending on the side (either left or right) which they are holding them. In this regard, a bias for holding infants on the left side of the body has been observed by most individuals—both adults (Malatesta, Marzoli, et al., 2019a) and children (Forrester et al., 2020)—probably due to a right-hemisphere advantage in promptly detecting potential cues of distress from the face of the cradled infant (Brosch et al., 2007; Colasante et al., 2017; Malatesta, Marzoli, et al., 2021b). Several studies have linked this “left-cradling bias” with perceptual and emotional biases in processing both adult (Bourne & Todd, 2004; Malatesta, Marzoli et al., 2021a) and infant (Huggenberger et al., 2009; Malatesta et al., 2020) faces. In addition, this bias might play a crucial role in the later development of the infant behaviour, a link having been hypothesized between early postural and lateral asymmetries during infancy and the ontogeny of the brain lateralization (Malatesta, Marzoli et al., 2021b; Michel & Harkins, 1986).

It must be noticed, moreover, that cradling and holding in arms are not the only occasions in which the infant head (and thus their face) assumes a noncanonical orientation with respect to that of the adult caregiver. Actually, it is more a rule than an exception that infants are seen from viewpoints that deviate from canonical (e.g., when the infant sleeps, is nursed, bathed). However, whereas these frequent activities are not likely to involve the expression of systematic left-right asymmetries of face-to-face alignment, cradling is acknowledged as a major lateral bias, and might have the potential to act as a postural condition influencing face perception (Parente & Tommasi, 2008). According to this view, in the present study we aimed to find out whether individuals exhibiting a left-cradling bias might show an advantage for processing infant emotional faces presented at an angle of rotation consistent with their lateral preference for holding infants (i.e., rotated clockwise 270° rather than 90°). In fact, when one cradles an infant on the left side of the body, the infant face—from the cradler’s point of view—turns out to be rotated clockwise ca. 270° (i.e., rotated anticlockwise 90°), possibly fostering a perceptual preference for such a specific angle of rotation compared with its opposite (i.e., rotated clockwise 90°). The existence of a potential link between postural and perceptual biases has been also suggested by a recent study in which left-cradling women were shown to judge as more attractive the left- rather than right-facing profile of an infant compared with right-cradling ones (Malatesta et al., 2020).

In sum, the aim of this study was to investigate, in a sample of female and male adults, the existence of potential biases in processing adult and infant faces using an emotion recognition task (i.e., detecting happy vs. sad expressions), also according to the angle of rotation at which they were presented. In particular, we hypothesized a general advantage in recognizing the emotional expression of adult rather than infant faces due to the own-age bias described in the literature of face recognition (Rhodes & Anastasi, 2012; H1), and a female advantage (in terms of better performance) in recognizing the emotional expression of both adult and infant faces, more evident for sad rather than happy faces of infants (Hampson et al., 2021; H2). Moreover, we hypothesized a stronger inversion effect for sad than happy adult faces (Calvo & Nummenmaa, 2008; H3), and an inversion effect for both sad and happy infant faces (H4). Finally, a left-cradling participants’ effect in recognizing the emotional expression of faces rotated consistently with their lateral preference for holding infants (i.e., rotated clockwise 270° instead of 90°), and vice versa for right-cradling participants, has been hypothesized (H5).

## Materials and methods

### Participants and stimuli

Using G*Power 3.1 (University of Kiel, GER), we conducted a power analysis to compute the required sample size to test our hypotheses by fixing the alpha at 0.05, power at 0.95, and an effect size based on a previous research on inverted emotional faces (Calvo & Nummenmaa, 2008). Accordingly, we planned to recruit a minimum of 128 participants. A total of 129 (62 female and 67 male) participants were included in this study. Their age ranged between 18 and 63 (*M* = 29.7, *SD* = 1.1) years and nine of them were left-handed (i.e., scored zero or negatively on the Italian version of the Edinburgh Handedness Inventory; Salmaso & Longoni, 1985; *M* = 76.2, *SD* = 47.4). Most of the participants were unmarried (*N* = 108) and nonparent (*N* = 93).

The stimuli used were pictures of human emotional faces: 10 (five male and five female) adult actors expressing both happy and sad emotional faces were extracted from the Karolinska Directed Emotional Faces database (KDEF; Goeleven et al., 2008); 10 (five male and five female) 4- to 12-month-old infant actors expressing both happy and sad emotional faces from the Tromso Infant Faces database (TIF; Maack et al., 2017). The pictures were converted into grey-scale images and an oval-shaped white mask was used to hide the hair to prevent participants’ identification of any potential diagnostic cues during the task. Each upright picture was rotated clockwise 90°, 180° (inverted) and 270° to obtain 4 angles of rotations. In addition, each picture was mirrored horizontally. All pictures were presented at a resolution of 347 × 418 pixels.

### Procedure

Data were collected remotely—due to the COVID-19 lockdown restrictions which prevented laboratory experiments—using E-Prime Go (Psychology Software Tools, Pittsburgh, PA). Through written electronic instructions, participants were invited to download the E-Prime Go application, to get in a quiet room with a balanced light source, to sit comfortably at around 57 cm from the centre of the computer screen placed centrally on an empty desk. Therefore, participants launched the application, and the experiment instructions were displayed on the screen. When participants were ready, they were required to start the experiment by a key press.

**Fig. 1:**
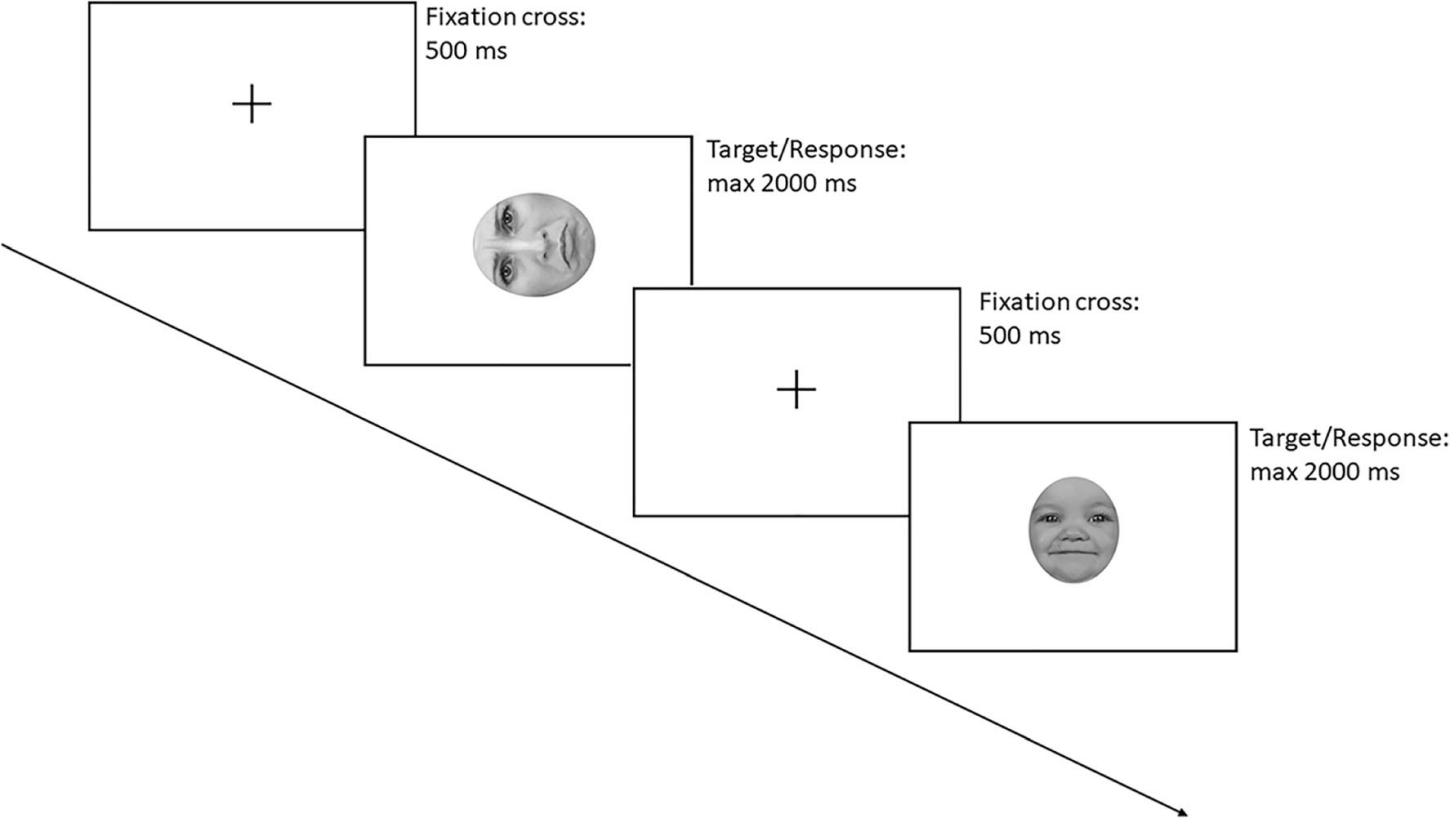
Example of two experimental trials (i.e., a happy sad adult face rotated 270° and an upright happy infant face)

The task consisted of 320 trials (divided into two blocks separated by a pause) in each of which a black fixation cross, presented for 500 ms at the centre of a white screen, was followed by the target stimulus (a sad or happy face) presented centrally for up to 2,000 ms. Participants were instructed to gaze at the fixation cross and to detect whether the face shown was expressing a happy or a sad emotion by pressing a key as fast and accurately as possible, within 2,000 ms, after which another trial began (see Fig. 1). Half of the sample had to press the key “G” to respond happy and the key “L” to respond sad, and vice versa for the other half of the sample. The measurement of the response time started with stimulus onset and stopped when the key was pressed.

Once the participant completed the experiment, he/she was required to fill out an online survey via Qualtrics (Qualtrics, Provo, UT) in which his/her lateral cradling preference (either left or right) was assessed using a single item stating: “Please imagine you are holding a newborn infant. Try to imagine their face (eyes, mouth, etc.) while you cradle them for a few seconds. On which side of your body did you imagine to hold the infant?” (Harris et al., 2000; Malatesta, Marzoli et al., 2019b; Nakamichi & Takeda, 1995).

### Data analysis

After a preliminary analysis showing strong positive correlations between response times and proportion of errors (see the Open Practices Statement), as suggested by Bruyer and Brysbaert (2013), we decided to use as dependent variable the Inverse Efficiency Score (IES) to provide a better summary of the findings (Townsend & Ashby, 1978). This index is computed by dividing response times by the proportion of correct responses in each condition (Townsend & Ashby, 1983). This means that the lower the IESs, the faster and more accurately the emotions were recognized (i.e., better performances), and vice versa.

We carried out a General Linear Model 2 × 4 × 2 × 2 × 2 analysis of variance (ANOVA) using Age Category of the faces (Adult vs. Infant), Rotation of the faces (0°, 90°, 180° and 270°) and Emotion of the faces (Happy vs. Sad) as within factors, and Sex of participants (Female vs. Male) and Cradling-side bias (Left vs. Right) as between factors. Before calculating the IESs, participants’ responses provided within 150 ms from the display of the target were excluded from the analysis. Moreover, the IESs that exceeded three standard deviations from the mean of each condition were removed before performing the analysis. Statistical analyses were performed using Statistica software package (StatSoft, Tulsa, OK). The significance threshold was set at *p* < .05 and Bonferroni test was used for all post hoc comparisons. All procedures and tools used here are based on up-to-date methodologies for understanding of cognitive and affective responses to stimuli in psychological research (Casado-Aranda et al., 2020).

## Results

According to participants’ responses to the lateral cradling preference survey, a different proportion of left- and right-cradlers, respectively, *N* = 86 (66.7%) vs. *N* = 43 (33.3%); χ^2^(1) = 14.33, *p* < .001, was observed. Specifically, a larger proportion of left- and right-cradlers was shown in both female, respectively, *N* = 41 (66.1%) vs. *N* = 21 (33.9%); χ^2^(1) = 6.45, *p* = .011, and male, respectively, *N* = 45 (67.2%) vs. *N* = 22 (32.8%); χ^2^(1) = 7.90; *p* = .005, participants.

ANOVA showed significant main effects of: Age Category (*M*_Adult_ = 736.25, *SEM*_Adult_ = 29.12; *M*_Infant_ = 957.31, *SEM*_Infant_ = 37.97), *F*(1, 109) = 588.60, *p* < .001, η_p_^2^ = 0.844, Rotation (*M*_0°_ = 779.77, *SEM*_0°_ = 22.99; *M*_90°_ = 841.47, *SEM*_90°_ = 22.65; *M*_180°_ = 928.85, *SEM*_180°_ = 27.60; *M*_270°_ = 837.03, *SEM*_270°_ = 25.19), *F*(3, 327) = 94.56, *p* < .001, η_p_^2^ = 0.464; all means differed from each other significantly, except for the 90° vs. 270° comparison; *p* = 1.000; Emotion (*M*_Happy_ = 869.85, *SEM*_Happy_ = 39.22; *M*_Sad_ = 823.70, *SEM*_Sad_ = 35.16), *F*(1, 109) = 10.42, *p* = .002, η_p_^2^ = 0.087, and Sex of participants (*M*_Female_ = 813.59, *SEM*_Female_ = 15.57; *M*_Male_ = 879.96, *SEM*_Male_ = 15.72), *F*(1, 109) = 9.00, *p* = .003, η_p_^2^ = 0.076.

**Fig. 2:**
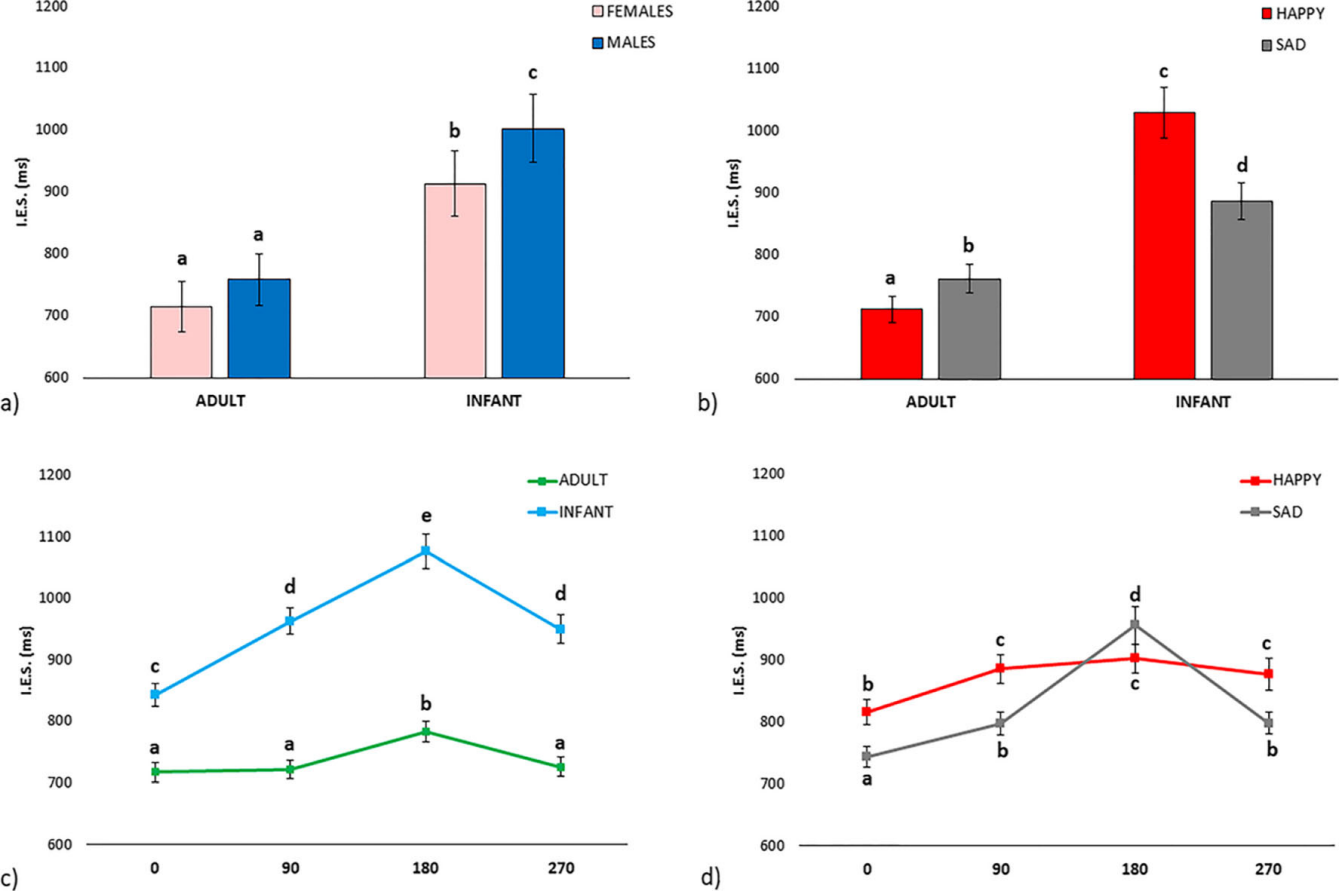
Significant double interactions: **a** Age Category × Sex of participants. **b** Age Category × Emotion. **c** Rotation × Age Category. **d** Rotation × Emotion. Different letters indicate significant differences (*p* < .05)

Moreover, four significant double interactions were shown: Age Category × Rotation, *F*(3, 327) = 39.30, *p* < .001, η_p_^2^ = 0.265; Age Category × Emotion, *F*(1, 109) = 79.15, *p* < .001, η_p_^2^ = 0.421), Rotation × Emotion, *F*(3, 327) = 19.64, *p* < .001, η_p_^2^ = 0.153; and Age Category × Sex of participants, *F*(1, 109) = 6.09, *p* = .015, η_p_^2^ = 0.053 (Fig. 2 shows post hoc differences for these interactions).

**Fig. 3:**
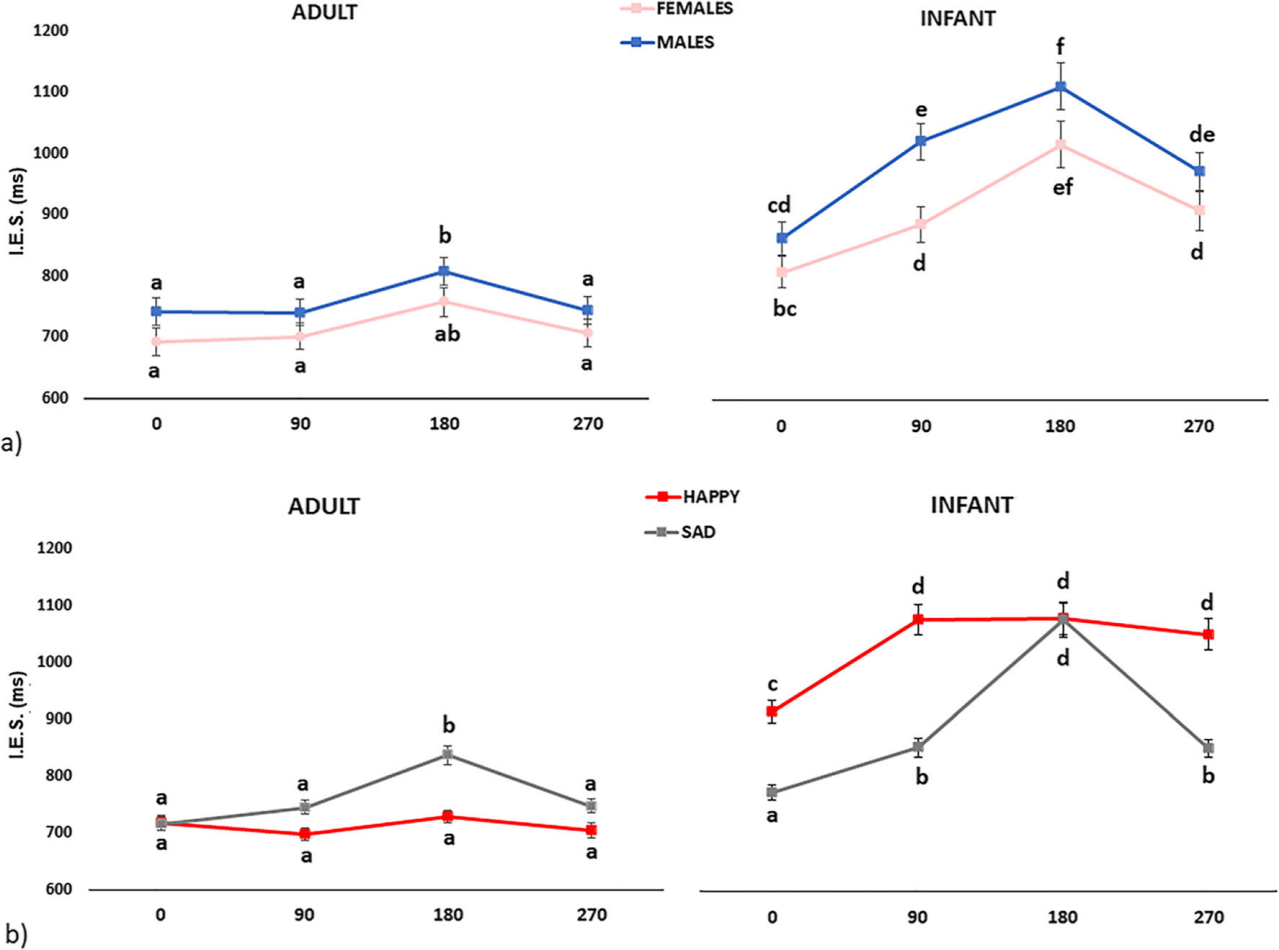
Significant triple interactions: **a** Age Category × Rotation × Sex of Participants. **b** Age Category × Rotation × Emotion. Different letters indicate significant differences (*p* < .05)

In addition, two triple interactions were shown: Age Category × Rotation × Sex of participants, F(3, 327) = 3.19, *p* = .024, η_p_^2^ = 0.028 (Fig. 3a) and Age Category × Rotation × Emotion, *F*(3, 327) = 9.2247, *p* < .001, η_p_^2^ = 0.078 (Fig. 3b). As regards the first interaction, post hoc analysis showed that Males (but not Females; *p* > .055) performed worse for 180° Adult faces than for all the other Rotations (all *p*s < .001). Females performed better than Males for 90° Infant faces (*p* = .048), but not for all the other Rotations (all *p*s > .907). Participants performed better for Adult than Infant faces regardless of the Rotations (all *p*s < .001). Finally, participants—regardless of Sex—performed better for 0° than 90°, 180°, 270° Infant faces (all *p*s < .001), with no difference between 90° and 270° (both *p*s > .646).

As regards the second interaction, post-hoc analysis showed that participants performed worse for 180° than 0°, 90° and 270° Sad Adult faces (all *p*s < .001), with no difference between all the Rotations of Happy Adult faces (all *p*s = 1.000). Moreover, participants performed worse for Happy Infant than Happy Adult faces for all the Rotations (all *p*s < .001); the same pattern occurred for Sad Infant than Sad Adult faces (all *p*s < .001), except for the 0° Rotation (*p* = .168). In addition, participants performed better for the 0° Sad Infant faces than all the other Rotations (*p* < .001), with no difference between 90° and 270° Rotations (*p* = 1.000). On the other hand, participants performed better for upright 0° than 90°, 180° and 270° Happy Infant faces (*p* < .001), with no differences between 90°, 180° and 270° Rotations (all *p*s = 1.000). Finally, participants performed better for Sad than Happy Infant faces regardless of the Rotations (all *p*s < .001) except for the 180° Rotation (*p* = 1.000).

## Discussion

The main aim of this study was to fill a gap in knowledge by using the face inversion paradigm in order to explore the adult attentional and perceptual systems during a task of emotion recognition of infant compared with adult faces. Furthermore, the existence of potential differences associated with specific postural biases (i.e., the left-cradling bias) during adult–infant interactions was investigated for the first time.

As expected, given that our sample consisted of adult participants, a bias for recognizing the emotion from own-age (adult) rather than other-age (infant) faces was confirmed (Rhodes & Anastasi, 2012; H1). Further, our data showed that females were better than males at recognizing the emotion (both happy and sad) from infant—but not adult—faces (H2), regardless of the emotion displayed. This finding is in line with previous studies in the domain of attention and perception suggesting a crucial role of the baby schema for reproductive success in humans (Thompson-Booth et al., 2014). In this regard, it has been suggested that the female expertise in processing of emotional infant faces might be associated with their role of primary caretakers assumed during evolution (Thompson & Voyer, 2014). Moreover, as regards the recognition of emotions from infant faces, better performances were found for sadness rather than happiness, regardless of participants’ sex. This result partially corroborates the “fitness threat hypothesis” according to which negative emotions expressed by infants represent a biologically salient stimulus for women (Hampson et al., 2021). However, we found this effect also in men and this can be due to the fact that infant sadness might represent a cue of the infant’s distress, the quick and accurate response to which—compared with happiness—could be crucial for the survival of the individuals, regardless of sex. Moreover, it should be noted that sex differences, in terms of a female advantage, in the processing of emotions from infant faces is not always observed (Thompson & Voyer, 2014), and a recent study found modest differences only limited to the facial disgust (Connolly et al., 2019).

As one can easily imagine, the main effect of face rotation revealed a general worsening of participants’ performances between the upright and the inverted conditions, with in-between performances for both intermediate rotations (which did not differ from one another). This is consistent with previous studies showing a linear relationship between angle of rotation and face recognition accuracy, although not in experiments involving emotions (Collishaw & Hole, 2002).

However, the significant interaction between age category, rotation and emotion (Fig. 3b) provided a detailed and explanatory overview of most of the results. As regards adult faces, it was shown that upside-down inversion did not influence the recognition of happiness, whereas it impaired the recognition of sadness, thus confirming that this mechanism is more based on the use of configural information for sad faces and of featural information for happy faces (Calvo & Nummenmaa, 2008; H3). Interestingly, although infant faces were generally processed worse than adult faces, results did not show any difference between the emotion recognition of upright infant faces expressing sadness and upright adult faces expressing sadness or happiness. This finding clearly highlights the social and biological salience of the sad infant face, even capable of overcoming the effectiveness of the own-age effect (Rhodes & Anastasi, 2012).

Whereas performances for intermediate rotations did not differ from the upright presentation as regards adult faces, emotion-dependent effects were found as regards infant faces (H4). For infant happiness, the performance worsening—compared with the upright presentation—was the same regardless of the angle of rotation; differently, for infant sadness, performance worsening was gradual, with both intermediate rotations falling in between the upright and the inverted presentations. Therefore, inversion seemed to have a larger influence on the emotion recognition of infant rather than adult faces following a linear relationship. In fact, this process seemed to take place earlier than with adults, manifesting already at both intermediate rotations. This finding suggests that emotion recognition of infant faces relies more on configural than featural information: it is possible to suppose that such a different processing is also due to the lack of some diagnostic facial cues that are present in the adult but not in the infant face. For instance, it has been found that when one smiles, exposed teeth produce a local contrasting luminance in the mouth area capable of catching the observer’s attention and thus facilitating the detection of happiness (Calvo & Nummenmaa, 2008). The presence of this and other diagnostic cues might be the reason why adult happiness is based on featural rather than configural emotion processing. On the other hand, the lack of similarly detectable cues on the infant face might trigger a configural rather than a featural mechanism, causing the worsened performances already observable at the intermediate rotations.

Finally, no relation between the lateral preference for holding infants and the performances for the two intermediate angles of rotations (i.e., rotated clockwise 270° instead of 90°) was found (H5). It is possible to conclude that the link between these postural and perceptual biases—as initially suggested—is not detectable by means of a modified version of the face inversion paradigm as used in this study. Because of the possible involvement of right-hemisphere networks in conveying the lateral cradling preference, further studies should investigate potential differences between left- and right-cradling individuals’ processing of emotional faces using a divided visual field paradigm (Bourne & Todd, 2004; Huggenberger et al., 2009). In this regard, the present study was conducted online (due to the COVID-19 lockdown restrictions), which is why participants’ lateral cradling preference was self-reported as part of a survey. Nevertheless, it should be noted that the imagery task is a reliable test for motor behaviours (Marzoli et al., 2017) such as cradling (Malatesta, Marzoli, et al. 2019a), even when administered to individuals without prior experience of interaction with infants (Harris et al., 2000; Nakamichi & Takeda, 1995). However, it is expected that further studies should directly assess participants’ cradling-side preferences (using a real infant or a life-like doll) in order to handle a more accurate evaluation of the bias.

The present study confirms that emotion processing of infant faces differs when compared with adult faces and further studies are needed to reach a more complete understanding of the cognitive mechanisms involved in the processing of this special category of stimuli.
